# Supplementary figures and images for: Lactate-Induced CCL8 in Tumor-Associated Macrophages Accelerates the Progression of Colorectal Cancer through the CCL8/CCR5/mTORC1 Axis
Source: Cancers (Basel). 2023 Dec 11;15(24):5795. doi: 10.3390/cancers15245795 (PMC10741879; doi:10.3390/cancers15245795)

Figure 2A

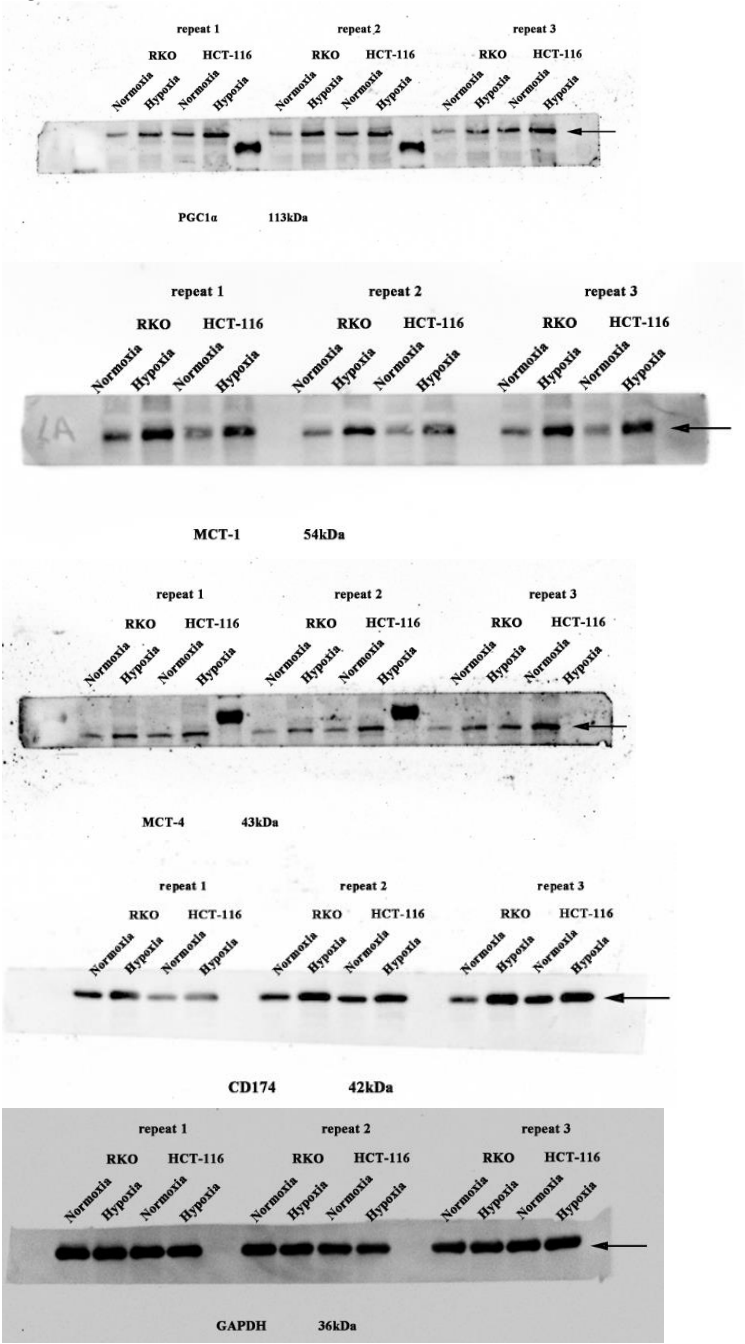

Figure 3D

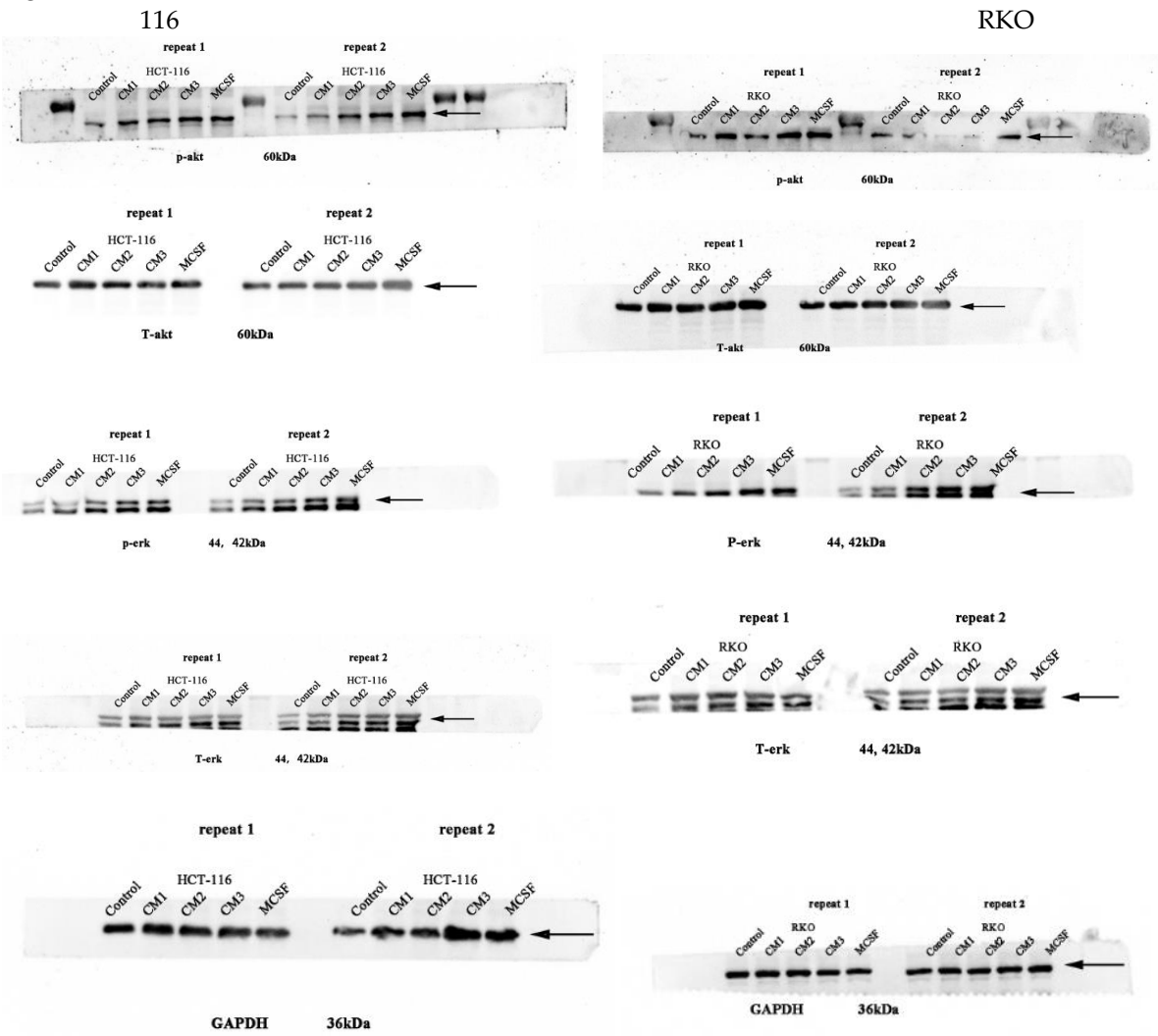

Figure 3E

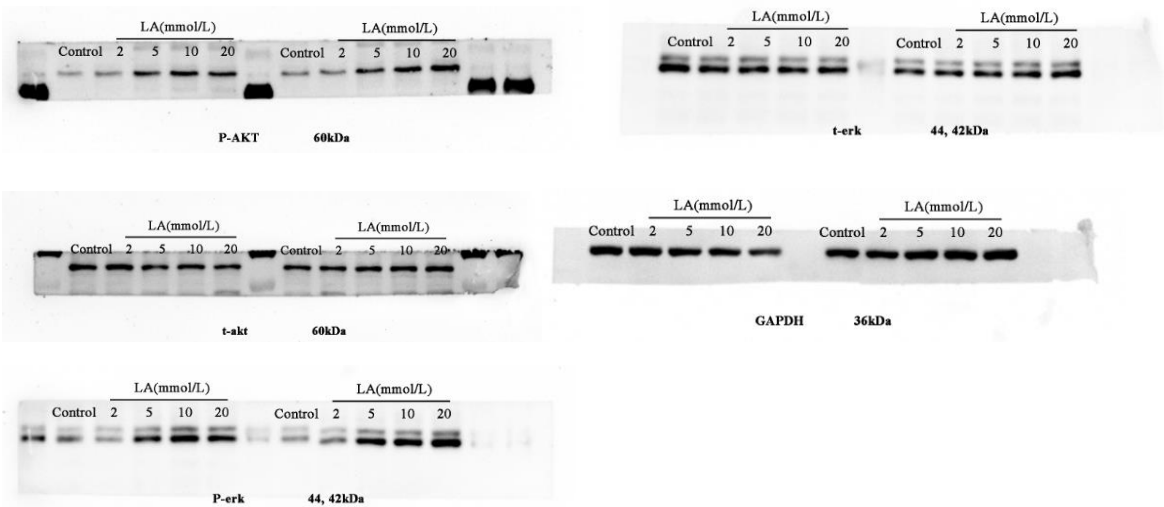

Figure 3G

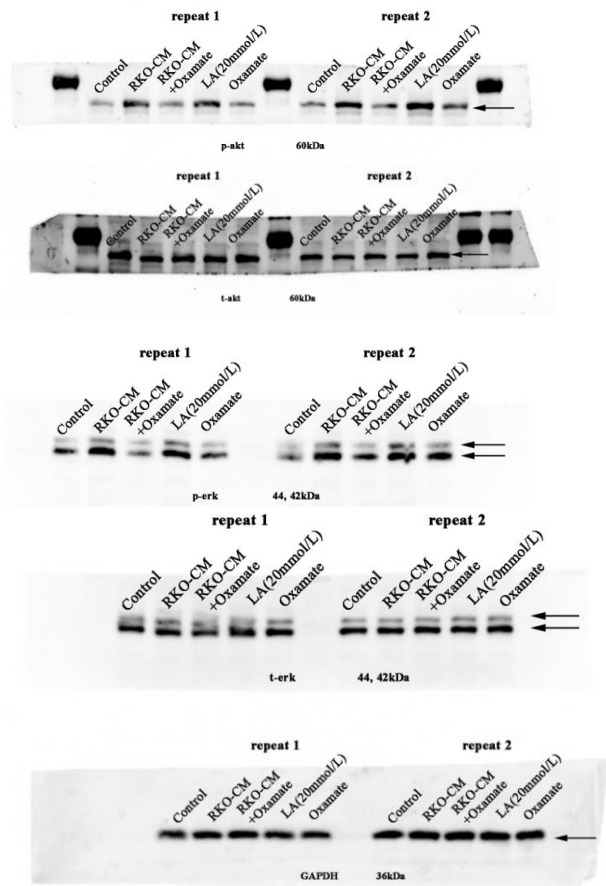

Figure 3J

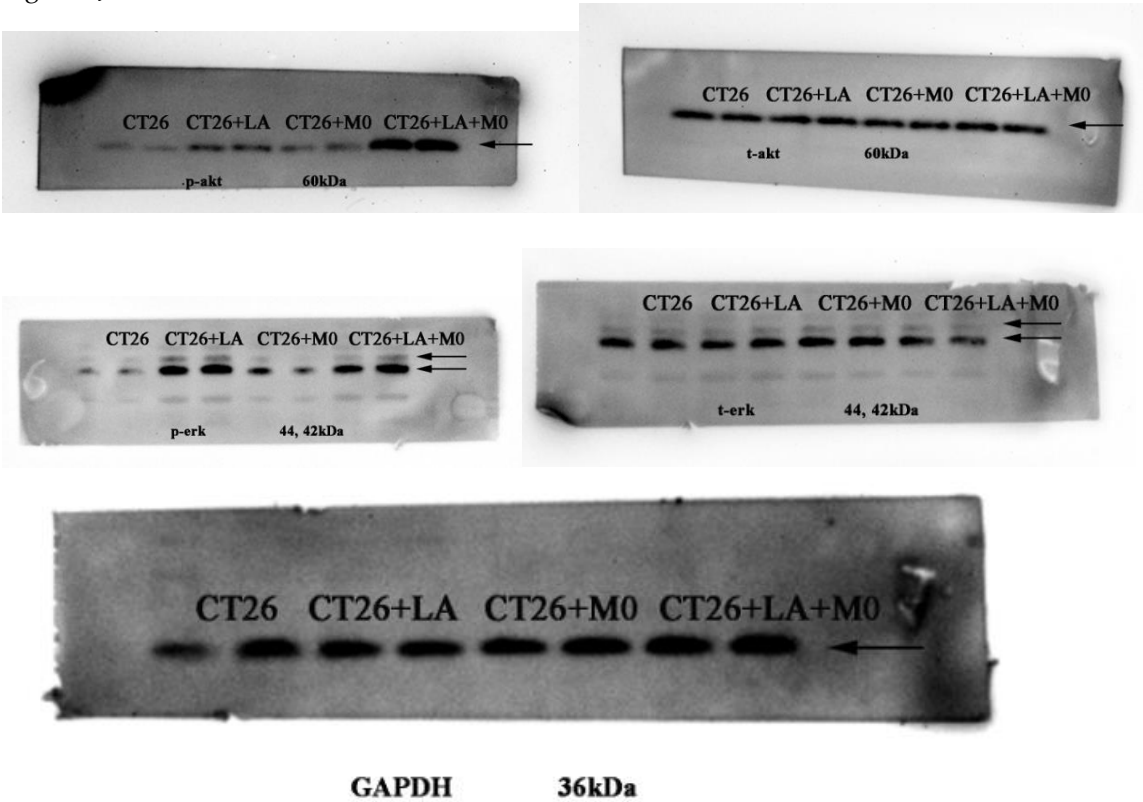

Figure 5A

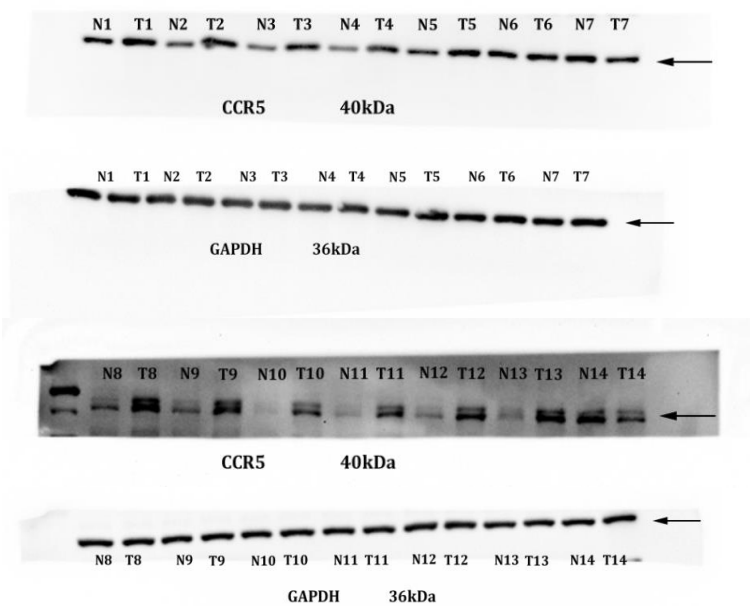

Figure 5F

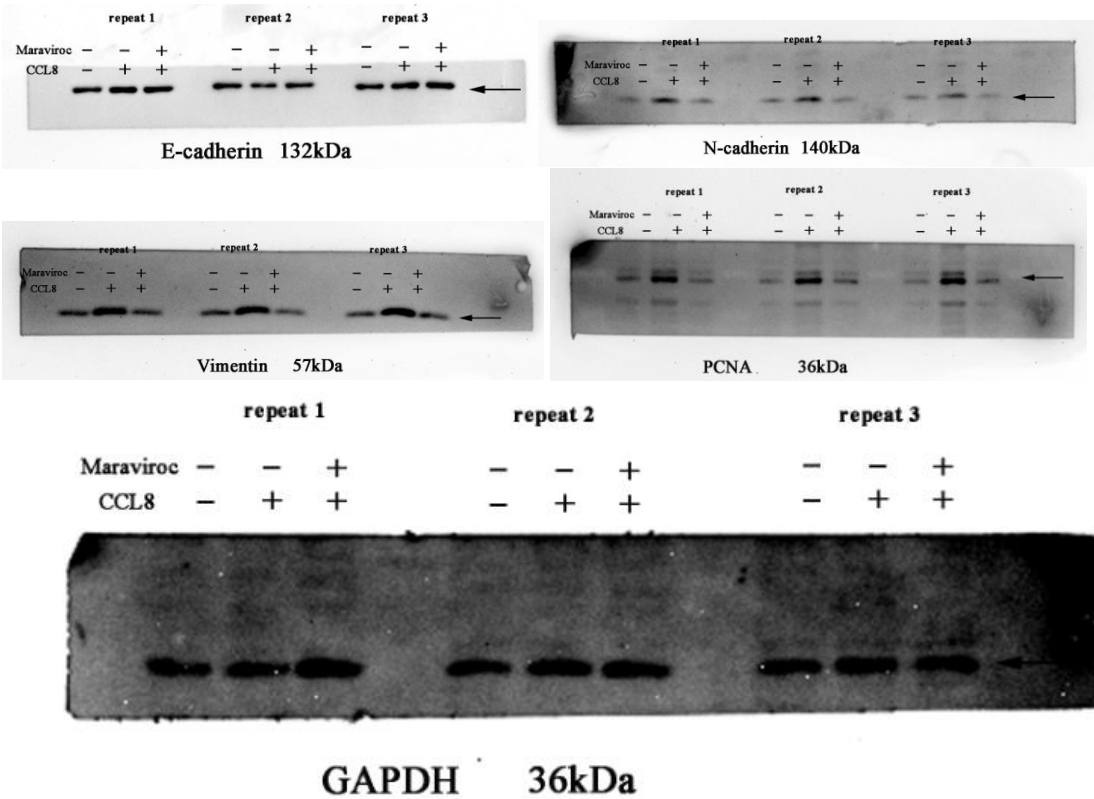

Figure 6A

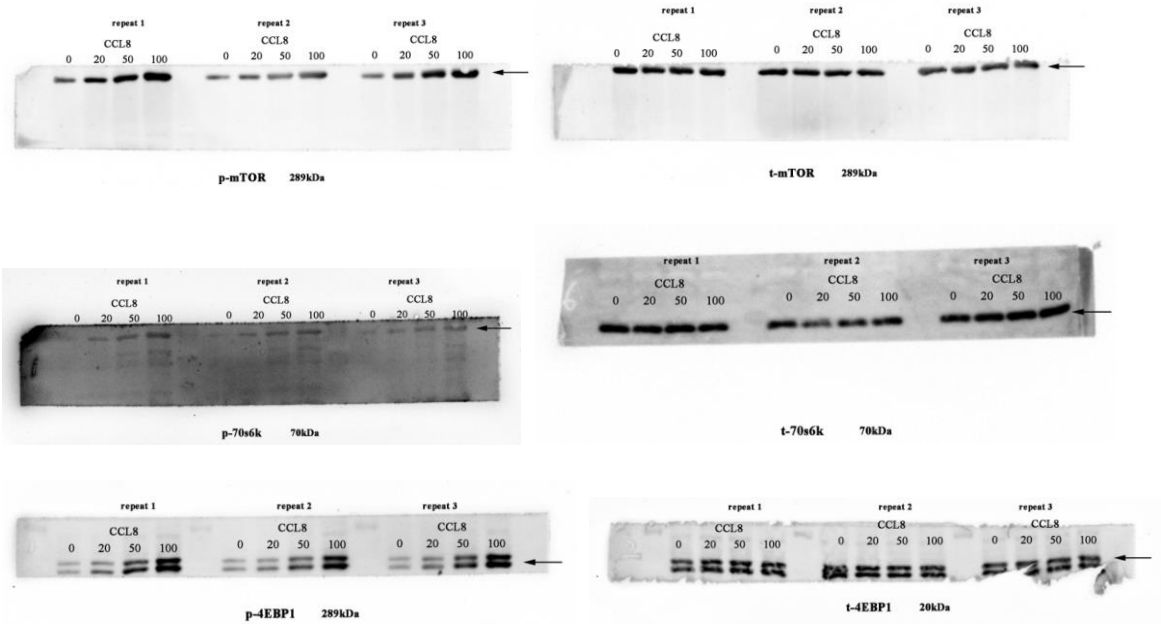

Figure 6B

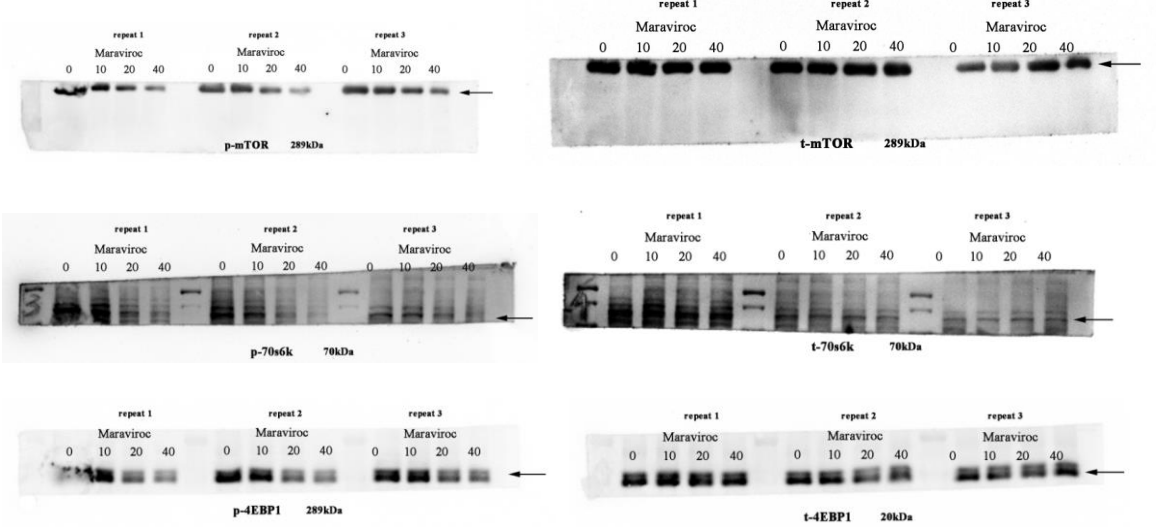

Figure 6C

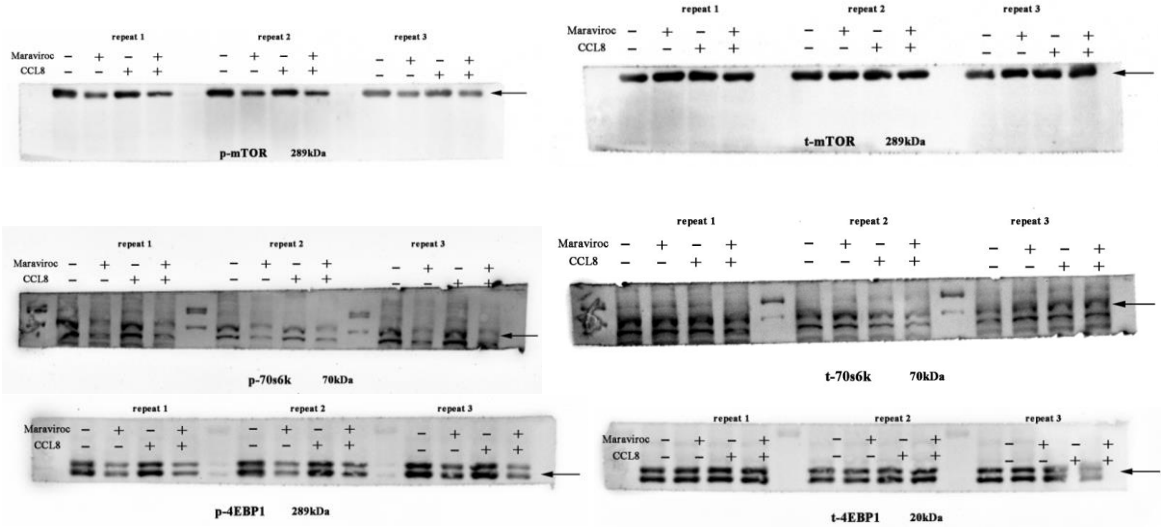

Figure 6D

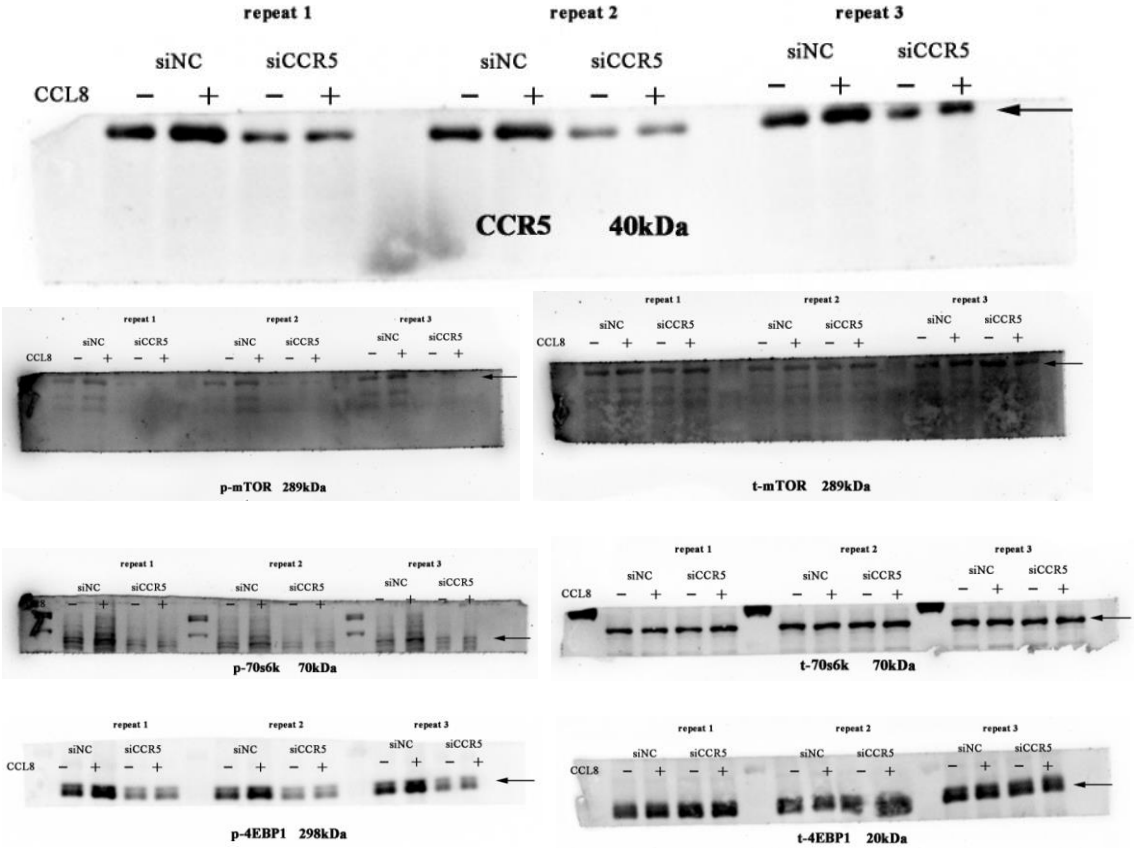

Supplement: Supplementary file 1 [file cancers-15-05795-s001.zip › cancers-2715900-supplementary.pdf]
